# The effect of infectious dose on humoral and cellular immune responses in *Chlamydophila caviae* primary ocular infection

**DOI:** 10.1371/journal.pone.0180551

**Published:** 2017-07-05

**Authors:** Ana Filipovic, Ehsan Ghasemian, Aleksandra Inic-Kanada, Ivana Lukic, Elisabeth Stein, Emilija Marinkovic, Radmila Djokic, Dejana Kosanovic, Nadine Schuerer, Hadeel Chalabi, Sandra Belij-Rammerstorfer, Marijana Stojanovic, Talin Barisani-Asenbauer

**Affiliations:** 1 Department of Research and Development, Institute of Virology, Vaccines and Sera – TORLAK, Belgrade, Serbia; 2 OCUVAC – Center of Ocular Inflammation and Infection, Laura Bassi Centres of Expertise, Center for Pathophysiology, Infectiology and Immunology, Medical University of Vienna, Vienna, Austria; University of Texas at San Antonio, UNITED STATES

## Abstract

Following infection, the balance between protective immunity and immunopathology often depends on the initial infectious load. Several studies have investigated the effect of infectious dose; however, the mechanism by which infectious dose affects disease outcomes and the development of a protective immune response is not known. The aim of this study was to investigate how the infectious dose modulates the local and systemic humoral and the cellular immune responses during primary ocular chlamydial infection in the guinea pig animal model. Guinea pigs were infected by ocular instillation of a *Chlamydophila caviae*-containing eye solution in the conjunctival sac in three different doses: 1×10^2^, 1×10^4^, and 1×10^6^ inclusion forming units (IFUs). Ocular pathology, chlamydial clearance, local and systemic *C*. *caviae*-specific humoral and cellular immune responses were assessed. All inocula of *C*. *caviae* significantly enhanced the local production of *C*. *caviae*-specific IgA in tears, but only guinea pigs infected with the higher doses showed significant changes in *C*. *caviae*-specific IgA levels in vaginal washes and serum. On complete resolution of infection, the low dose of *C*. *caviae* did not alter the ratio of CD4^+^ and CD8^+^ cells within guinea pigs’ submandibular lymph node (SMLN) lymphocytes while the higher doses increased the percentages of CD4^+^ and CD8^+^ cells within the SMLN lymphocytes. A significant negative correlation between pathology intensity and the percentage of CD4^+^ and CD8^+^ cells within SMLN lymphocyte pool at selected time points post-infection was recorded for both 1×10^4^, and 1×10^6^ IFU infected guinea pigs. The relevance of the observed dose-dependent differences on the immune response should be further investigated in repeated ocular chlamydial infections.

## Introduction

Almost six million people are blind or visually impaired due to trachoma caused by the obligate intracellular bacterium *Chlamydia trachomatis* (Ct) serovars A–C, which is the most common infectious cause of blindness worldwide [1–4]. In trachoma-endemic communities, the prevalence of ocular Ct infection decreases with age, and the highest bacterial loads are found in young children, suggesting that a degree of protective immunity develops following natural infection [5]. The clinical outcomes of ocular Ct infection range from no inflammation/disease to severe and sight-threatening sequelae, raising questions on the nature of host-pathogen interactions. The key question is why only a minority of people living in trachoma-endemic regions develop severe scarring complications. The likely explanation is the interplay between the lifetime burden of infection among individuals and their local immune response. The immunological basis of scarring trachoma is not well understood; whether it is driven primarily through cell-mediated adaptive or epithelial cell-derived innate responses is unclear [6]. Moreover, the balance between protective immunity and immunopathology may depend on the initial infectious load. It has already been shown that Ct dose affects the balance of B-/T-cell responses *in vitro* [7].

Inclusion conjunctivitis, an ocular infection caused by *Chlamydophila caviae* in guinea pigs, is a well-characterized and accessible model for studying trachoma [8, 9]. *C*. *caviae* infection in guinea pigs closely resembles the disease process of ocular Ct infection in humans [10]. Guinea pigs are naturally infected with the chlamydial species, *C*. *caviae*. Murray first isolated *C*. *caviae* from the infected conjunctivae of young laboratory guinea pigs and defined it as the causative agent of guinea pig inclusion conjunctivitis [11]. The infection of guinea pigs with human Ct serovars D and E [12], and the usage of this model for Ct vaccination studies [13], was described in the genital, but not in the ocular, animal model. The major disadvantage of the ocular guinea pig model has been the lack of a wide range of immunological reagents/consumables, knockout animals, and easily accessible inbred guinea pig strains. Recently, a novel guinea pig gene expression RT-qPCR array was developed, which might advance the utilisation of the guinea pig model and help to better our understanding of the immune responses after infection/immunisation with Chlamydiae [14].

Researchers were able to characterise important aspects of disease progression and protection in the guinea pig ocular model, mostly in repeated infections: i) complete or marked reduction in the intensity of infection upon reinfection [11, 15], ii) the development of cell-mediated immunity demonstrating that a trachoma-like disease could be elicited by repeated infections [16], and iii) enhancement of the local and serum antibody responses against Chlamydiae as a result of infection and reinfection [17]. In our previous study, we examined the effect of infectious dose on host response in repeated infections [18].

The effect of infectious dose on ocular infection kinetics and the resulting pathologic responses using the guinea pig ocular inclusion conjunctivitis model [8, 19] were described in primary ocular chlamydial infection [8], but no profiling of immune responses after the onset of infection was performed. Lacy et al. used primary ocular infection with *C*. *caviae* to investigate whether acute inflammation had a role in modulating the adaptive immune response [20], although the experiments were performed using only one dose: 1×10^4^ inclusion-forming units (IFU). The study successfully determined the essential role of neutrophils in the immunopathology of primary chlamydial infections.

The importance of infectious dose was also proven in other chlamydial animal models. It has been demonstrated that earlier onset of infection is dose dependent; death of the subject at higher doses and survival at lower doses for respiratory infections with *C*. *muridarum* have been reported [21]. It has also been shown that the infectious dose of *C*. *muridarum* affects both the rate and extent of clearance and ascension in the female reproductive tract, as well as the development of gross pathology [22]. Furthermore, the infectious dose of *C*. *muridarum* modulates the innate immune response and ascending infection [23]. Earlier onset of infection with increased peak levels of organisms in guinea pig infections with *C*. *caviae* have also been reported [24]. Recent studies involving calves intra-bronchially infected with different doses of *C*. *psittaci* resulted in dose-dependent pulmonary and systemic host reactions, ranging from mild to severe forms [25]. Intranasal infection with a low/medium dose of *C*. *abortus* in non-pregnant sheep results in a latent infection that leads to infection of the placenta and abortion in a subsequent pregnancy. In contrast, a high dose stimulates protective immunity, resulting in a much lower abortion rate [26].

Doses used so far in studies investigating the immune responses in primary ocular chlamydial infection models of 1×10^4^ IFU and above were shown to induce exaggerated ocular pathology. It is not known whether these doses are relevant in the “real life” situation. In the guinea pig genital chlamydial infection model the natural infectious dose was determined as 1×10^2^ IFU [24]. Therefore, we infected guinea pigs with a 10^2^ IFU dose to induce ocular infection and compared low (1×10^2^), moderate (1×10^4^) and high (1×10^6^) doses to determine the effect of the infectious dose on immune response vs. pathology in primary ocular chlamydial infection.

## Materials and methods

### Ethics statement

The experiments were approved by the ‘‘Ethics Committee for the Welfare of Experimental Animals” at the Institute of Virology, Vaccines and Sera–Torlak, conformed to the Serbian laws and European regulations on animal welfare (Approval No. 323-07-01577/2016-05/12), and adhered to the Association for Research in Vision and Ophthalmology Statement for the Use of Animals in Ophthalmic and Vision Research. All animals were handled in strict accordance with good animal practice as defined by the Serbian code of practice (published in Službeni Glasnik No. 41/9) for the care and use of animals for scientific purposes, the Guide for the Care and Use of Laboratory Animals of the Torlak Institute (2133/1, 21. 04. 2011), and the Basel Declaration that is committed to the 3R principle (replace, reduce, refine). Animal testing was planned and carried out with extreme care. Every effort was made to minimise animal suffering. The guinea pigs were observed daily by trained animal care staff, and animals requiring care were referred to the attending veterinary surgeon for immediate care. Terminal euthanasia was carried out by lethal CO_2_ overdose. We did not observe any unexpected deaths of animals during this study, nor were there any severe incidents that required euthanasia of an animal.

### Experimental animals

Female Hartley strain guinea pigs weighing 300–350 g, and 6 weeks of age were used in this study. Each group included 5 animals (5 animals per group for each infectious dose at the defined time point), with 85 animals in total. Animals were housed individually in cages with filter tops, given food and water *ad libitum*, and kept on a 12-h light/12-h dark cycle. The guinea pigs were pre-screened by in-house optimised enzyme-linked immunosorbent assay (ELISA) as previously described [18].

### *Chlamydia* strain and conjunctival infections

**Fig 1:**
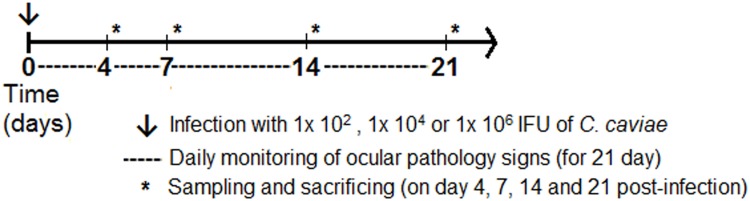
Experimental design. Guinea pigs were infected on day 0 via inoculation of 1×10^2^, 1×10^4^ or 1×10^6^ IFUs of *C*. *caviae* directly into the conjunctival sac (arrow). Pathological signs were followed daily for 21 days post-infection (dashed line). Days 4, 7, 14 and 21 post-infection are chosen as time points when sampling of mucosal washes, sera and swabs were performed, and *ex vivo* analyses were performed (asterisks).

*C*. *caviae*, guinea pig inclusion conjunctivitis strain, was kindly provided by Prof. Roger G. Rank, prepared in his laboratory through continuous passage, first in yolk sac and then in tissue culture. Stocks of *C*. *caviae* were produced according to standard methodology [27] in McCoy cells and frozen at -80°C in sucrose-phosphate-glutamate (SPG) buffer until needed. The animals were anaesthetized intramuscularly with a mixture of ketamine (30 mg/kg) and xylazine (2 mg/kg). Inoculation was performed on day 0 on the anaesthetised guinea pigs by instilling 25 μl of SPG buffer containing 1×10^2^, 1×10^4^, and 1×10^6^ IFU of *C*. *caviae* directly into the conjunctival sac with a micropipette, while the control group received SPG buffer only. Only one eye of each animal was inoculated with *C*. *caviae*. The doses were chosen according to previously established criteria: i) high dose (1×10^6^ IFU) ensures aggravated ocular disease [28], ii) moderate dose (1×10^4^ IFU) ensures 100% infection in all animals, with a pathology response that can be easily quantified by gross observation [20], and iii) low dose (1×10^2^ IFU) approximates natural genital infection in guinea pigs [24]. These three doses of *C*. *caviae* have previously been used to induce ocular infection in guinea pigs, and there have been no reports of visual disability [8, 19]. Our experimental procedure did not cause any visual impairment in the guinea pigs, and visual examinations were continually performed over the post-infection period. None of the applied doses caused changes in behavioural patterns in the treated animals relative to their respective controls or disrupted their normal daily activity. The guinea pigs were sacrificed on days 4, 7, 14 and 21 post-infection (abbreviated as dpi4, dpi7, dpi14, and dpi21, respectively). Blood, conjunctival swabs, tears, vaginal washes, and lymph nodes were taken for further analysis. The schedule of monitoring and sample collection during the post-infection period is described in Fig 1.

### Pathology scoring

A trained ophthalmologist who was blinded to the experimental groups examined the guinea pigs’ eyes daily by visual scoring of gross ocular pathology. The palpebral and bulbar conjunctivae were evaluated for erythema, oedema, and exudation in each animal. Each observation was classified into 5 categories: (0.5) trace pathologic response, (1) slight erythema or oedema of either the palpebral or bulbar conjunctiva, (2) definite erythema or oedema of either the palpebral or bulbar conjunctiva, (3) definite erythema or oedema of both the palpebral and bulbar conjunctiva, or (4) definite erythema or oedema of both the palpebral and bulbar conjunctiva plus the presence of exudate.

### Isolation of *Chlamydia* from conjunctival swabs

Conjunctival swabs for the isolation and quantification of *C*. *caviae* were collected from the guinea pigs while under ketamine/xylazine anaesthesia. Conjunctival swabs were collected with the Copan Universal Transport Medium (UTM-RT) System (Copan Italia, Brescia, Italy) and frozen at -80°C until needed. The numbers of IFU were determined by culture in McCoy cells, as previously described [29]. In brief, McCoy cell monolayers were infected with swab material using centrifugation, and the infection was stopped after 24 hours with 100% methanol fixation. *C*. *caviae* IFUs were visualised by staining with a fluorescein isothiocyanate (FITC)-conjugated monoclonal antibody against chlamydial lipopolysaccharide (Clone B410F, Pierce Biotechnology, Rockford, IL, USA). IFUs were counted using a fluorescence microscope (Axio Observer Zeiss, Vienna, Austria).

### Myeloperoxidase immunohistochemistry

Immunohistochemical staining for myeloperoxidase, a neutrophil-specific enzyme, was performed on 3 μm paraffin-embedded conjunctival sections. A rabbit polyclonal antibody to myeloperoxidase (Sigma-Aldrich, Germany), followed by anti-rabbit IgG-biotin (Sigma-Aldrich, Germany) and ExtrAvidin^®^−Peroxidase (Sigma-Aldrich, Germany) were used. Detection was performed with 3,3′-diaminobenzidine (DAB) (DAKO, Denmark).

### Determination of *C*. *caviae*-specific antibody response

Peripheral blood samples were taken from the lateral saphenous veins while the guinea pigs were under anaesthesia. Serum samples, tears, and vaginal washes were collected on day 0 (prior to each infection) and on dpi4, dpi7, dpi14, and dpi21. ELISA was performed to quantify the *C*. *caviae* specific IgG levels in serum samples. To measure *C*. *caviae* specific IgA levels in samples, ELISA plates (MaxiSorp^™^) (Nalge Nunc International, Roskilde, Denmark) were coated (50 μl/well) with Renografin-purified *C*. *caviae* elementary bodies (EB; 1×10^6^ EBs/ml in SPG buffer) and incubated overnight at 4°C. The next day, 2% (w/v) bovine serum albumin in phosphate-buffered saline (BSA/PBS) was added as a blocking reagent for 2 h at a room temperature (RT) of 21°C. This blocking step and all subsequent ELISA steps were followed by a wash with 0.05% (v/v) Tween-20 in PBS (four times, 200 μl/well). Sera (1:100), tears (1:24), and vaginal washes (1:24) from individual animals were each diluted in 1% (w/v) BSA/PBS and added to the wells in duplicate. The samples were incubated on the plates for 2 h at 37°C. Antigen-specific antibody binding was detected after a 1-h incubation at RT using anti-guinea pig IgA (MyBioSource, San Diego, CA, USA) at a 1:800 dilution in 1% (w/v) BSA/PBS. Furthermore, a dilution of 1:20,000 of peroxidase-conjugated anti-sheep IgG antibody (Sigma-Aldrich, St. Louis, MO, USA) in 1% (w/v) BSA/PBS was added to each well. After a 1-h incubation at RT, the antigen-antibody interactions were visualised using 3,3',5,5'-tetramethylbenzidine (TMB) substrate (eBioscience, San Diego, CA, USA). Colour development was stopped within 30 min by adding 2 M sulphuric acid, and absorbance values were measured at 450 and 650 nm.

### Proliferation assay

Submandibular lymph node (SMLN) cells were isolated and prepared, as previously described, [30] and stimulated with 1×10^6^ IFU/ml live *C*. *caviae* EBs. Cell Counting Kit-8 reagent (CCK8) (Sigma-Aldrich, Germany) was used to quantify the number of viable cells in proliferation according to the manufacturer’s instructions. In brief, 10 μl/well of CCK8 was added after 48 h of incubation, and the cells were incubated for a further 4 h. The reaction was stopped by the addition of 1% (w/v) sodium dodecyl sulphate (10 μl/well), and absorbance values were measured at 450/650 nm (A_450/650_) using a spectrophotometer (Ascent 6–384 [Suomi]) (MTX Lab Systems Inc., Vienna, VA, USA). The number of viable cells per well was calculated using a standard curve A_450/650_ = f (number of cells). A discrete pool of non-stimulated cells (the viable cells count determined by trypan blue dye exclusion) was measured using an Invitrogen^™^ Countess^™^ Automated Cell Counter and used to produce a standard curve. A proliferation index (PI) for each specifically stimulated cell suspension was calculated for each animal. Presuming that stimulation did not affect the transformation rate of CCK8 and that number of viable cells was changed upon stimulation due to cell proliferation, the PI index was calculated as the ratio of the number of viable cells per well present in stimulated (Ss) cultures to the number of viable cells per well present in the corresponding non-stimulated (So) cultures (PI = Ss/So).

### Flow cytometry

SMLN cells (1×10^6^ cells/sample) were immunostained using fluorochrome-conjugated antibodies specific for guinea pig CD4 (PE-conjugated, BioRad-AbDSerotec) and CD8 (FITC-conjugated, BioRad-AbDSerotec). Prior to staining, the cells were washed with a cold 2% BSA/0.1% NaN_3_/PBS solution (2× centrifugation at 300 g, 5 min, 4°C). Fluorochrome-conjugated antibodies were added to the resuspended cell pellets and incubated in the dark for 30 min at 4°C. Discrete aliquots of each analysed cell suspension were incubated with the corresponding isotype control antibodies and used as an unstained reference for setting the analysis staining thresholds. Unbound antibodies were removed by washing in cold 2% BSA/0.1% NaN_3_/PBS solution (3× centrifugation at 300 g, 5 min, 4°C). Lymphocytes were gated per their position within forward scatter vs. side scatter plots and analysed for the percentage of CD4^+^ and CD8^+^ cells using the BD FACSVerse^™^ flow cytometer (BD Biosciences). BD CellQuest^™^ Pro software was used for analysis.

### Statistics

The statistical significance of the observed differences was evaluated using a two-way ANOVA test followed by Tukey’s multiple comparisons test. A bivariate Pearson correlation analysis was used to analyse the correlation. All statistical analyses were performed with GraphPad Prism 6.0 (GraphPad Inc., La Jolla, CA, USA). A probability (P) value of 0.05 was set as the significance threshold.

## Results

### Infectious dose influences the dynamics of pathology during primary *C*. *caviae* infection

**Fig 2:**
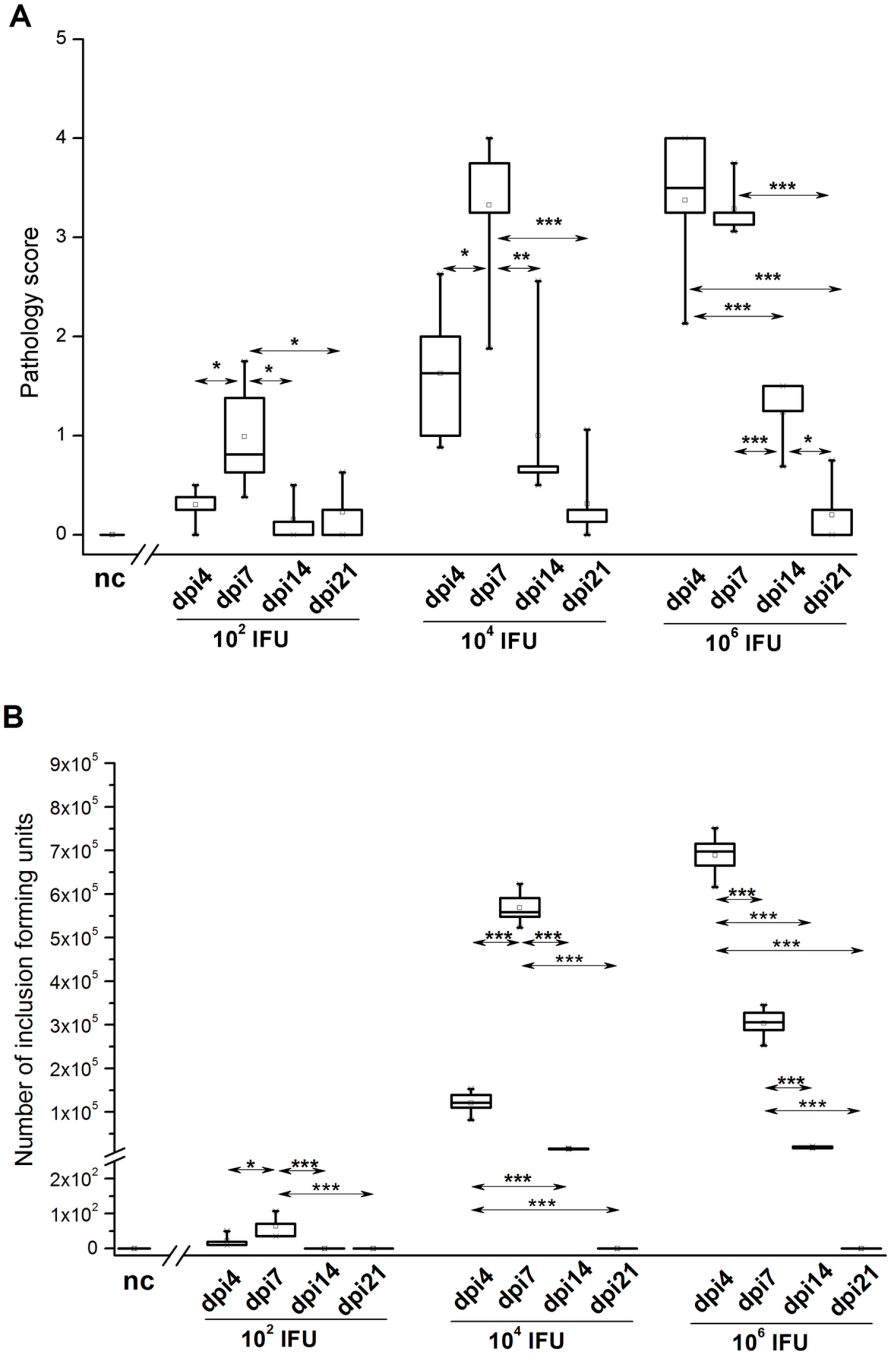
Ocular pathology scores (A) and *C*. *caviae* load (B) in guinea pigs infected with a single ocular instillation of three different *C*. *caviae* doses. The amount of *C*. *caviae* applied per guinea pig eye (expressed as IFU) and screening time points within the post-infection period (day 4 –dpi4, day 7 –dpi7, day 14 –dpi14, and day 21 –dpi21) are indicated on the x-axis. The start of infection is considered as day 0. Age-matched non-infected guinea pigs (nc) were used as negative controls. Statistical significance of the observed differences was evaluated using two-way ANOVA test followed by Tukey’s multiple comparisons test. The statistical significance of differences in parameter values between specific time points (compared groups indicated with arrows) for guinea pigs infected with an equal amount of *C*. *caviae* is indicated as follows: * P<0.05, ** P<0.005, and *** P<0.001.

Monitoring of ocular pathology in guinea pigs infected by the topical inoculation of *C*. *caviae* at three different doses revealed that the infectious dose influences the intensity of the pathology and the dynamics of pathology development. In guinea pigs inoculated with 1×10^2^ and 1×10^4^ IFU, pathology scores peaked at dpi7 and were significantly higher compared to the pathology scores recorded for corresponding (inoculated with an equal dose) animals at any other time point (Fig 2A). Furthermore, the pathology scores recorded on dpi4 and dpi7 for guinea pigs inoculated with 1×10^4^ IFU were significantly higher than those recorded at the same time points for guinea pigs inoculated with 1×10^2^ IFU (dpi4 P<0.05; dpi7 P<0.001).

In guinea pigs inoculated with 1×10^6^ IFU, an intensive ocular pathology was marked earlier (on dpi4) compared to those inoculated with 1×10^2^ and 1×10^4^ IFU (pathology scores on dpi4: 1×10^6^ IFU vs. 1×10^2^ IFU, P<0.001; 1×10^6^ IFU vs. 1×10^4^ IFU, P<0.05). Furthermore, pathology scores recorded in the guinea pigs inoculated with 1×10^6^ IFU on dpi4 and dpi7 were not significantly different from those recorded in guinea pigs inoculated with 1×10^4^ IFU on dpi7.

Bivariate Pearson’s correlation analysis showed a significantly positive correlation between the *C*. *caviae* burden (Fig 2B) and pathology severity (Fig 2A; Pearson’s correlation coefficient (Pcc) = 0.857, P<0.001). The correlation was most prominent in guinea pigs inoculated with 1×10^4^ and 1×10^6^ IFU (1×10^2^ IFU: Pcc = -0.192, P>0.05; 1×10^4^ IFU: Pcc = 0.853, P<0.001; 1×10^6^ IFU: Pcc = 0.824, P<0.001).

### Influx of neutrophils into the conjunctiva and conjunctiva-associated lymphoid tissue after *C*. *caviae* infection is dose dependent

**Fig 3:**
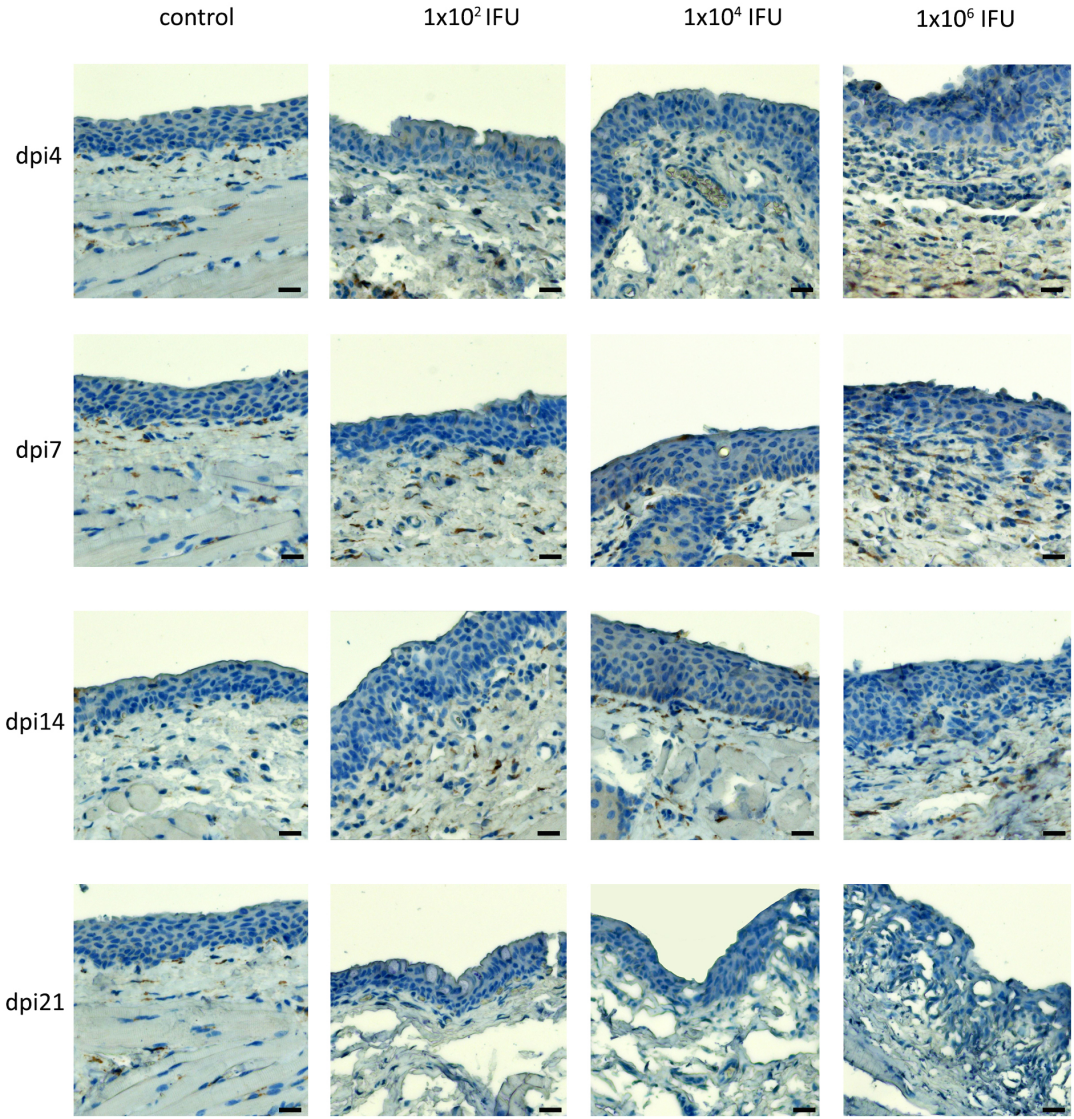
The abundance of neutrophils in the conjunctiva and CALT of guinea pigs infected with a single ocular instillation of three different *C*. *caviae* doses. The neutrophils infiltration was evaluated in paraffin-embedded conjunctival sections by immunohistochemical staining for myeloperoxidase, a neutrophil-specific enzyme. Conjunctival sections were prepared from samples collected at screening time points within the post-infection period (day 4 –dpi4, day 7 –dpi7, day 14 –dpi14, day 21 –dpi21). ExtrAvidin^®^−Peroxidase/DAB system was used for visualisation of myeloperoxidase presence.

A dose-dependent increase in neutrophil infiltration into the conjunctival tissue was evident in the infected animals (Fig 3). Guinea pigs inoculated with 1×10^4^ and 1×10^6^ IFU showed more neutrophils in the deeper layers of the conjunctiva and conjunctiva-associated lymphoid tissue (CALT). The strongest neutrophil infiltration of the conjunctival tissue was observed on dpi4 in animals inoculated with 1×10^6^ IFU. At lower infectious doses, more neutrophils were observed on dpi7. The overall damage to the conjunctival epithelium in the infected animals was comparable for 1×10^6^ and 1×10^4^ IFU inoculated animals and more severe in these two groups compared to the 1×10^2^ IFU inoculated group. Non-infected animals showed normal conjunctival morphology throughout the experiment. A comparison of changes in the number of neutrophils within infected conjunctiva and CALT (Fig 3) with changes in pathology severity post-infection (Fig 2A), showed that the influx of neutrophils was positively correlated with pathology scores.

### *C*. *caviae* infectious dose affects levels of IgA-mediated humoral immune response

**Fig 4:**
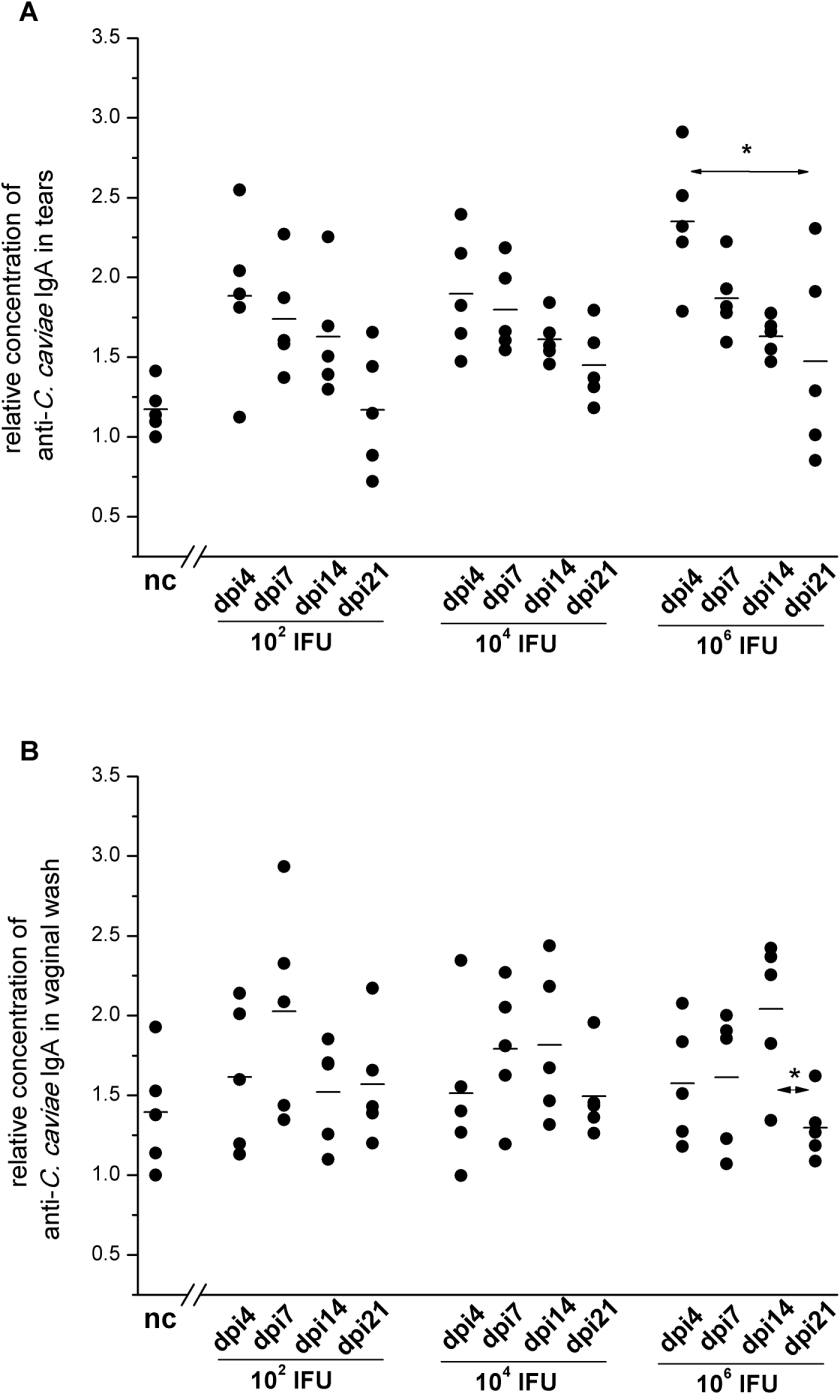
The levels of *C*. *caviae*-specific IgA in tears (A) and vaginal washes (B) of guinea pigs infected with a single ocular instillation of three different *C*. *caviae* doses. The amount of *C*. *caviae* applied per guinea pig eye (expressed in IFU) and screening time points within the post-infection period (day 4 –dpi4, day 7 –dpi7, day 14 –dpi14, day 21 –dpi21) are indicated on the x-axis. The start of infection is considered as day 0. Age-matched non-infected guinea pigs (nc) were used as negative controls. The level of specific antibodies is expressed as a *relative concentration*, calculated as the A_450/650_ for a sample divided by the lowest A_450/650_ recorded within the same assay for the corresponding nc sample. The statistical significance of the observed differences was evaluated using the two-way ANOVA test followed by Tukey’s multiple comparisons test. The statistical significance of the differences in parameter values between specific time points (compared groups indicated with arrows) for guinea pigs infected with an equal amount of *C*. *caviae* is indicated as follows: * P<0.05, ** P<0.005, and *** P<0.001.

The changes in *C*. *caviae*-specific IgA levels for different infectious doses of *C*. *caviae* were examined in tears (Fig 4A) and in vaginal washes (Fig 4B). All used inoculation doses significantly enhanced the local production of *C*. *caviae*-specific IgA (Fig 4A). The maximum local production of *C*. *caviae*-specific IgA was recorded on dpi4 (vs. negative control (nc): P<0.05 for 1×10^2^ IFU and 1×10^4^ and P<0.005 for 1×10^6^ IFU) and gradually decreased during the post-infection follow-up period (vs. nc: on dpi7 and dpi14 P<0.05, and on dpi21 P>0.05 for all infected groups). *C*. *caviae*-specific IgA levels in vaginal washes were found to be significantly elevated during the post-infection period only in guinea pigs inoculated with 1×10^6^ IFU (Fig 4B). The highest concentration of *C*. *caviae*-specific IgA in vaginal washes from 1×10^6^ IFU inoculated guinea pigs was recorded on dpi14. IgA levels from the animals inoculated with 1×10^6^ IFU of *C*. *caviae* were significantly higher than IgA levels in vaginal washes from the control non-infected animals (P<0.05) and vaginal washes taken on dpi14 from animals inoculated with 1×10^2^ IFU of *C*. *caviae* (P<0.05).

**Fig 5:**
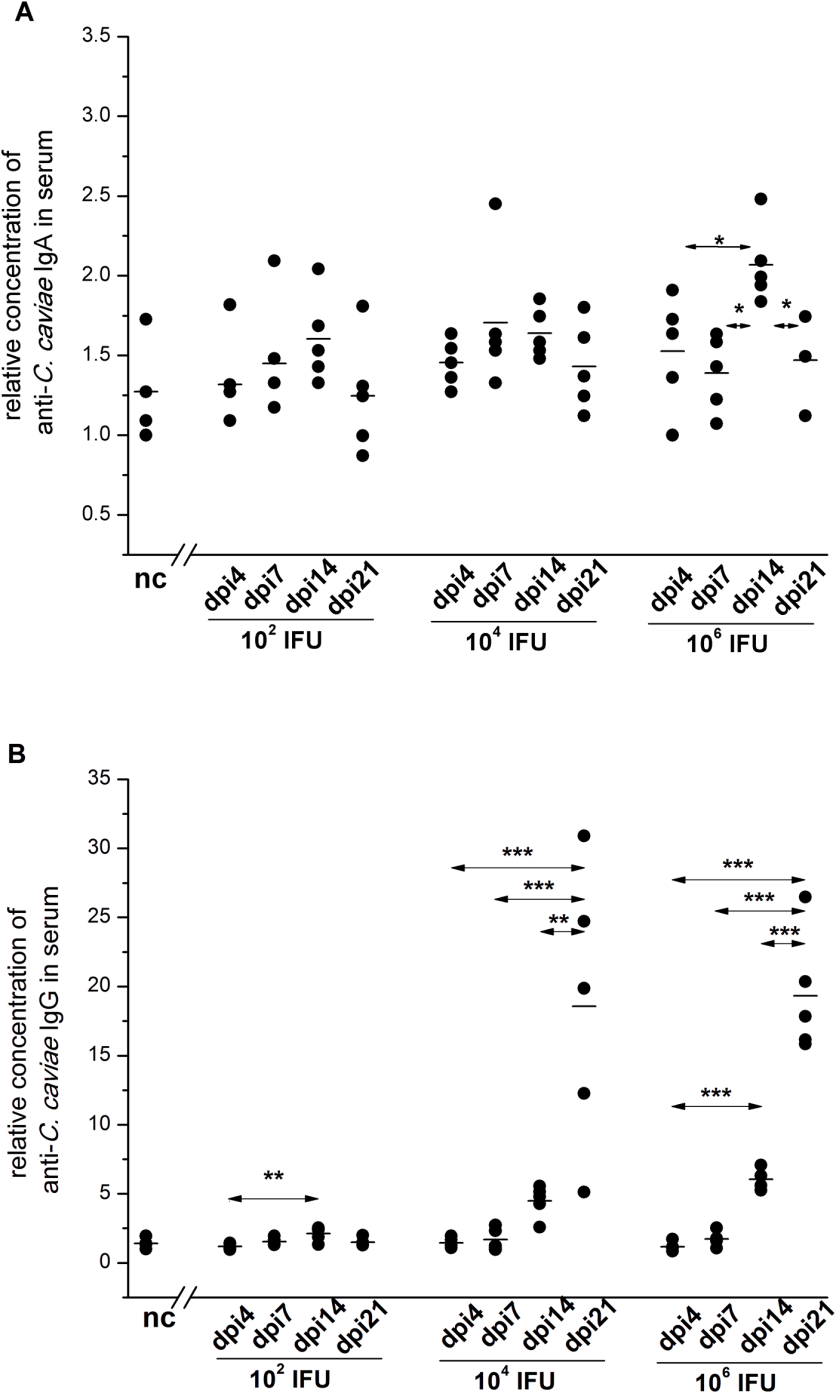
The levels of *C*. *caviae*-specific IgA (A) and IgG (B) in the sera of guinea pigs infected with a single ocular instillation of three different *C*. *caviae* doses. The amount of *C*. *caviae* applied per guinea pig eye (expressed in IFU) and screening time points within post-infection period (day 4 –dpi4, day 7 –dpi7, day 14 –dpi14, and day 21 –dpi21) are indicated on the x-axis. The start of infection is considered as day 0. Age-matched non-infected guinea pigs (nc) were used as negative controls. The level of specific antibodies is expressed as a *relative concentration*, calculated as the A_450/650_ for a sample divided by the lowest A_450/650_ recorded within the same assay for the corresponding nc sample. The statistical significance of the observed differences was evaluated using the two-way ANOVA test followed by Tukey’s multiple comparisons test. The statistical significance of differences in parameter values between specific time points (compared groups indicated with arrows) for guinea pigs infected with an equal amount of *C*. *caviae* is indicated as follows: * P<0.05, ** P<0.005, and *** P<0.001.

The sera of infected guinea pigs were also analysed for levels of *C*. *caviae*-specific antibodies, IgA and IgG. Our results revealed that a greater variation in *C*. *caviae*-specific antibody levels was associated with higher inoculation doses. The rise of *C*. *caviae*-specific IgA levels in the serum, up to dpi14, was statistically significant only in guinea pigs inoculated with 1×10^6^ IFU (Fig 5A). The levels of *C*. *caviae*-specific serum IgA recorded on dpi14 were significantly higher for guinea pigs inoculated with 1×10^6^ IFU than for those inoculated with lower doses of *C*. *caviae* (1×10^2^ IFU and 1×10^4^ IFU, P<0.05).

A significant rise in the levels of *C*. *caviae*-specific IgG in the sera of all infected guinea pigs was present on dpi14 (Fig 5B); the highest levels of *C*. *caviae*-specific IgG were recorded in the sera of guinea pigs inoculated with 1×10^6^ IFU of *C*. *caviae* (dpi14: 1×10^6^ IFU vs. 1×10^2^ IFU, P<0.001; 1×10^6^ IFU vs. 1×10^4^ IFU, P<0.05; 1×10^4^ IFU vs. 1×10^2^ IFU, P<0.005). The rise of *C*. *caviae*-specific IgG level in the sera of the guinea pigs inoculated with 1×10^4^ IFU and 1×10^6^ IFU continued after dpi14 with the highest level being recorded at the end of the follow-up period (dpi21: 1×10^6^ IFU vs. 1×10^4^ IFU, P>0.05).

### Responsiveness to *C*. *caviae* stimulation varies during the post-infection period but is not significantly influenced by the *C*. *caviae* infectious dose

**Fig 6:**
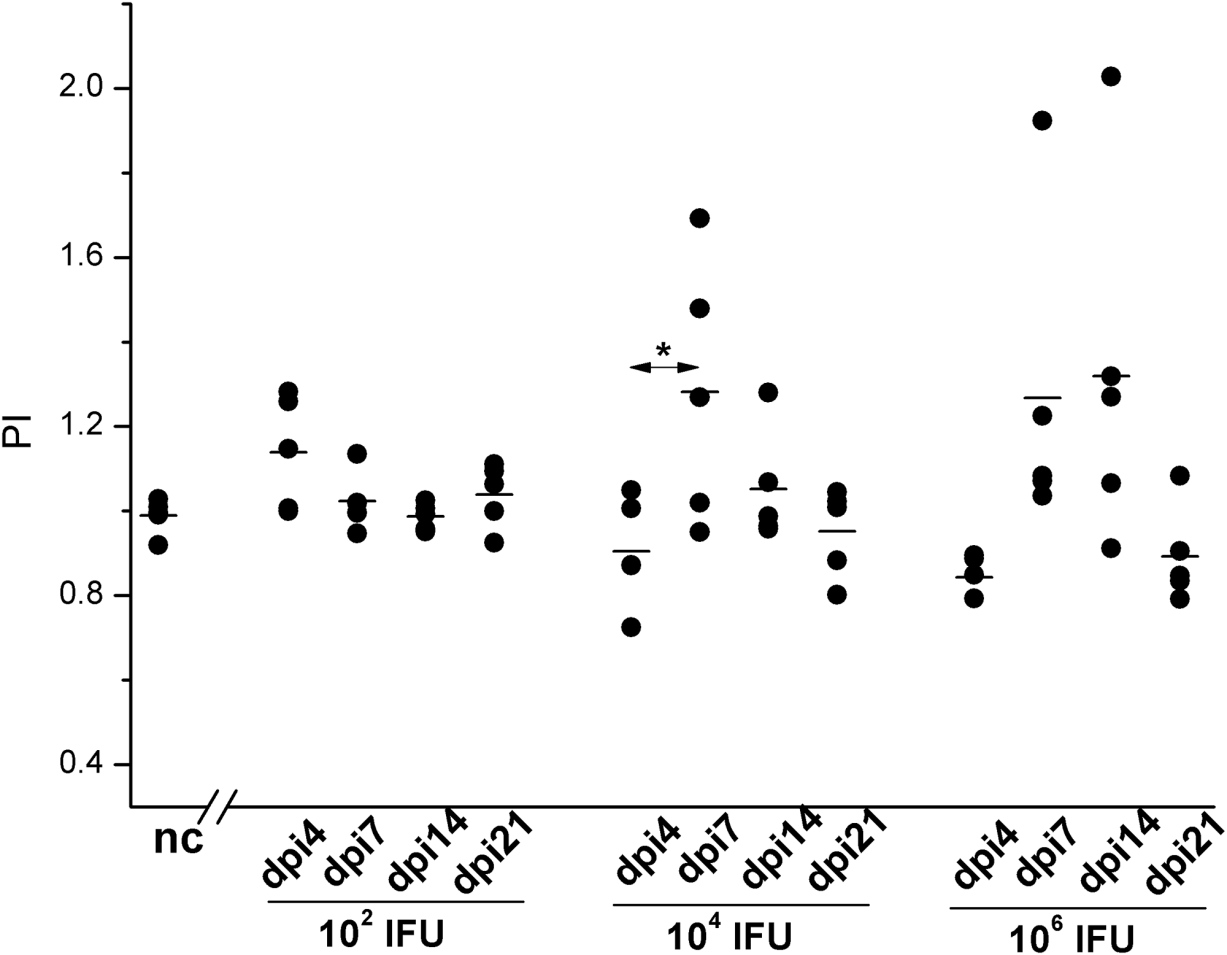
Proliferation of SMLN cells after stimulation with live *C*. *caviae*. Cell cultures were prepared from SMLN and spleen cells collected at defined time points (post-infection: day 4 –dpi4, day 7 –dpi7, day 14 –dpi14, and day 21 –dpi21) from guinea pigs infected with *C*. *caviae* (infectious doses per eye, expressed in IFU, assigned on x-axes) and age-matched non-infected guinea pigs (nc; used as negative controls). The start of infection is considered as day 0. The PI is calculated per individual sample and defined as a ratio of number of viable cells per well present in a stimulated culture to the number of viable cells per well present in the corresponding non-stimulated culture. The statistical significance of the observed differences was evaluated using the two-way ANOVA test followed by Tukey’s multiple comparisons test. The statistical significance of the differences in parameter values between specific time points (compared groups indicated with arrows) for guinea pigs infected with an equal amount of *C*. *caviae* is indicated as follows: * P<0.05, ** P<0.005, and *** P<0.001.

The proliferative response analyses of SMLN cells (Fig 6) collected on dpi4 from the guinea pigs inoculated with 1×10^2^ IFU revealed no significant increase in their responsiveness in comparison with corresponding cells collected from non-infected control guinea pigs. Furthermore, compared to the SMLN cells from the 1×10^2^ IFU inoculated guinea pigs, the responsiveness of SMLN cells to *C*. *caviae in vitro* stimulation was lower for both the 1×10^4^ IFU (P<0.05) and 1×10^6^ IFU (P<0.05) inoculated guinea pigs during the early post-infection period (on dpi4). Bivariate Pearson’s correlation analysis performed for all guinea pigs infected with *C*. *caviae* showed a negative correlation between the pathology scores recorded on dpi4 and proliferative responses to *C*. *caviae in vitro* stimulation recorded for the corresponding (from the same animal) SMLN taken at the same time point (pathology score vs. SMLN PI: Pcc = -0.667, P = 0.001). SMLN cells collected from the 1×10^4^ IFU and 1×10^6^ IFU inoculated guinea pigs on dpi7 showed a greater proliferation after stimulation with *C*. *caviae* compared to the corresponding cultures of cells collected on dpi4 (P<0.05 for 1×10^4^ IFU inoculated guinea pigs). After dpi7, the SMLN cell responsiveness from the 1×10^4^ IFU inoculated guinea pigs to *in vitro* stimulation with *C*. *caviae* gradually decreased. In the 1×10^6^ IFU inoculated guinea pigs the SMLN cell responsiveness on dpi7 and dpi14 remained at the same level and then dropped to the nc values on dpi21.

### Variations in the numbers of CD4^+^ and CD8^+^ cells within SMLN lymphocytes during the post-infection period depends on *C*. *caviae* infectious dose

**Fig 7:**
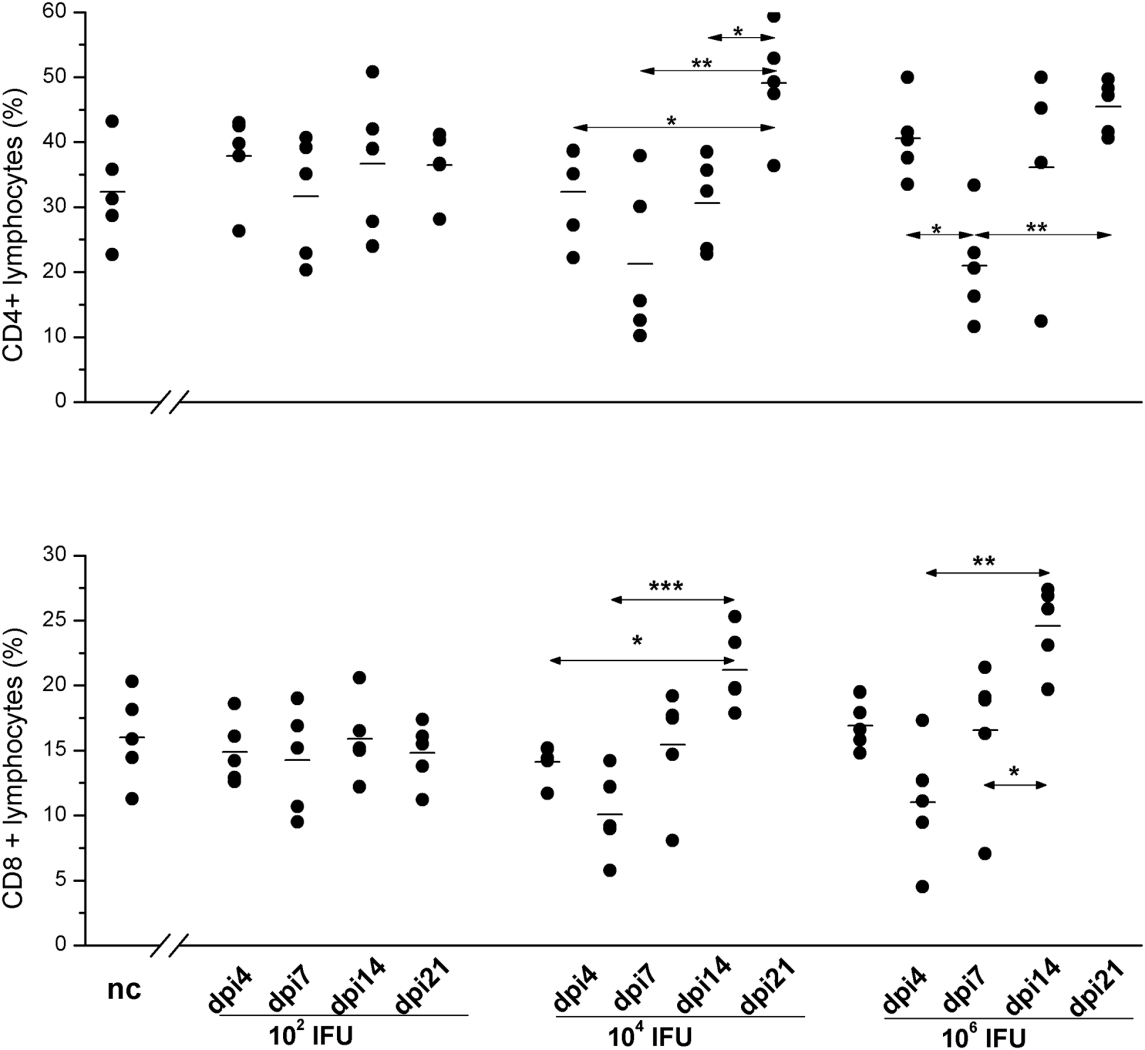
Expression of CD4 and CD8 on SMLN lymphocytes. Cell suspension for BD FACSVerse^™^ analyses were prepared from SMLN and splenic lymphocytes collected at defined time points (post-infection: day 4 –dpi4, day 7 –dpi7, day 14 –dpi14, and day 21 –dpi21) from guinea pigs infected with *C*. *caviae* (infectious doses per eye, expressed in IFU, assigned on the x-axis) and age-matched non-infected guinea pigs (nc; used negative controls). The start of infection is considered as day 0. The expression of CD4 and CD8 are presented as percentages of CD4^+^ and CD8^+^ cells within lymphocyte gates, respectively. Lymphocytes were gated according to their position within the forward scatter vs. side scatter plot. The statistical significance of the observed differences was evaluated using the two-way ANOVA test followed by Tukey’s multiple comparisons test. The statistical significance of the differences in parameter values between specific time points (compared groups indicated with arrows) for guinea pigs infected with an equal amount of *C*. *caviae* is indicated as follows: * P<0.05, ** P<0.005, and *** P<0.001.

Analysis of the percentage of CD4^+^ and CD8^+^ cells in SMLN lymphocytes infected with *C*. *caviae* via the ocular mucosa revealed no significant variation in the 1×10^2^ IFU inoculated animals (Fig 7). However, there were significant changes in the percentages of CD4^+^ and CD8^+^ cells in the guinea pigs inoculated with 1×10^4^ IFU and 1×10^6^ IFU, and the pattern alterations were comparable for both doses. The lowest percentages of CD4^+^ and CD8^+^ lymphocytes in the SMLNs of 1×10^4^ IFU and 1×10^6^ IFU inoculated guinea pigs were recorded on dpi7. Compared to the samples collected from non-infected control guinea pigs, the decrease in the percentages of CD8^+^ cells within the SMLN lymphocyte population was statistically significant for both groups (P<0.05) while the reduction in the percentage of CD4^+^ lymphocytes was not statistically significant in either group.

The highest percentages of both CD4^+^ and CD8^+^ within the SMLN of the 1×10^4^ IFU and 1×10^6^ IFU inoculated guinea pigs were recorded upon complete resolution of infection, on dpi21, and were significantly higher (P<0.05) compared to those of non-infected controls. Furthermore, over the whole post-infection period there was a negative correlation between pathology intensity and percentages of CD4^+^ and CD8^+^ within SMLN lymphocyte pool for both 1×10^4^ IFU (pathology score vs. CD4^+^ Pcc = -0.598, P = 0.005; pathology score vs. CD8^+^ Pcc = -0.679, P = 0.001) and 1×10^6^ IFU inoculated (pathology score vs. CD4^+^ Pcc = -0.462, P<0.05; pathology score vs. CD8^+^ Pcc = -0.637, P<0.005) guinea pigs.

## Discussion

We have demonstrated using three different doses of *C*. *caviae* that the infectious dose affects the levels of specific IgA-mediated humoral immune response as well as the changes in the percentages of CD4^+^ and CD8^+^ cells within SMLN lymphocytes during the post-infection period in an ocular guinea pig model.

Consistent with previous studies [8, 19], we have shown that the *C*. *caviae* dose influences the kinetics of chlamydial infection and the resulting gross pathologic findings with moderate (1×10^4^ IFU) and low (1×10^2^ IFU) doses, increasing the time to the peak of infection.

It is known that B cells play an important role in immunity to chlamydial infection in the mouse [31] and guinea pig model of chlamydial genital infection [32, 33]. The role of IgA in protective immunity is supported by *in vitro* studies showing the neutralising capabilities of anti-chlamydial antibodies [34–38] and *in vivo* data showing anti-chlamydial IgA in tears and vaginal washes [39–41] post-infection. In this study, ocular *C*. *caviae* infection significantly enhanced the production of *C*. *caviae*-specific IgA in tears with all inoculation doses.

The local increase in *C*. *caviae*-specific IgA levels occurred at the beginning of the post-infection period, on dpi4, and was transient. A period of 4 days after infection is insufficient for the development of specific class switched primary response, and these findings may be explained by the polyclonal activation of B cells locally present under physiological conditions [7, 42]. As with other infections [20, 43, 44], a rapid rise in *C*. *caviae*-specific IgA levels may imply stimulation of B cells already present in CALT to secrete antibodies that, due to cross-reactivity [45], recognise chlamydial antigens. Therefore, the IgA response to *C*. *caviae* may be affected by conjunctival B cells stimulated by ocular surface microbiome to develop a pre-infection pool of B cells cross-reactive with *C*. *caviae* proteins.

As the beginning of the post-infection period is characterised by the development of an inflammation-mediated pathology, also present in the 1×10^2^ IFU per eye inoculated dose, we hypothesised that the initial decrease in *C*. *caviae*-specific IgA production resulted from the inflammatory milieu due to neutrophil influx. It has been postulated that neutrophils may downregulate IgA humoral responses in ocular chlamydial infections, most likely by downregulating TGF-β and IL-5 as both are increased when neutrophils are depleted, and both are required for IgA production [20]. However, we were not able to test this hypothesis as the cytokine consumables for the guinea pigs were unavailable.

The kinetics of systemic IgA- and IgG-mediated immune responses with moderate and high infectious doses are indicative of an antigen-driven process and the development of specific adaptive immunity. Furthermore, if we take into account that specific IgG in tears is most likely to be derived from a systemic response [30, 46], the reduction in specific IgA local concentration prior to infection resolution may be compensated for by specific IgG production.

In addition to the appearance of chlamydial antigen-specific antibodies, another immunologic sign of chlamydial infection is the promotion of a proliferative response within the draining lymph node cells upon *in vitro* chlamydial stimulation [47]. Not surprisingly, on dpi4 there were no proliferative responses in SMLN cells collected from guinea pigs infected with all three infectious doses. Since these are primary cellular responses within 4 days of the infection, SMLN cells were either in an unresponsive phase very close to initial stimulation, or there had been insufficient time for a primary expansion. The *C*. *caviae*-specific proliferative responses detected in draining SMLN cells obtained on dpi7 and dpi14 for medium and high doses, but not for low doses, imply the development of specific immune response. It seems that low infectious doses are not sufficiently effective to trigger the specific cellular immune response.

A number of studies highlight the role of CD8^+^ cells in response to chlamydial infection [48–53]. It has also been suggested that local anti-chlamydial CD8^+^ cytotoxic T lymphocytes may be important in the resolution of naturally acquired human ocular chlamydial infections [54]. Furthermore, studies on animal models have shown that local CD4^+^ cell response via the secretion of IFNγ and stimulation of other protective immune cells is required for a successful resolution of chlamydial infection [55, 56].

In our experiment, unlike moderate and high *C*. *caviae* doses, low doses did not significantly alter CD4^+^ and CD8^+^ cells within guinea pig SMLNs. These findings also suggest that inoculation with 1×10^2^ IFU of *C*. *caviae* is less effective in the activation of the cellular immune response. This may be explained by the model proposed by Levitt et al. [7], suggesting that local or systemic infection with relatively low numbers of *Chlamydia* may result in stimulation of suppressor T cells, which can reduce both B cell proliferation and T helper cell activity and lead to down-regulation of the initial immune response. Moreover, responses to antigens presented during these periods may either be augmented (during early infection) or depressed (during later infection). The significant, albeit not strong, negative correlation between pathology intensity and percentage of CD4^+^ and CD8^+^ cells in the lymphocyte pool of SMLNs was recorded for moderate and high but not for the low chlamydial infectious doses. The beginning of the resolution of infection on dpi7 coincided with a significant drop in the frequency of CD4^+^ and CD8^+^ lymphocytes in draining lymph nodes implying that the efflux of activated CD4^+^ and CD8^+^ lymphocytes may be one of the explanations for the temporary drop in the percentage of CD4^+^ and CD8^+^ lymphocytes within the SMLN.

IFNγ, one of the key cytokines of Th1 responses, is important in the control of chlamydial infections via several well-described mechanisms [57]. It has been shown that peripheral blood mononuclear cells from individuals where trachoma is endemic proliferate and produce IFNγ, in response to chlamydial antigens, and these responses were weaker in subjects with trachomatous scarring when the antigens were absent [58]. Furthermore, IFNγ is increased in the conjunctivae of affected individuals during active chlamydial infection [59].

Although antigen-specific IFNγ has been shown to be critical for protection against chlamydial infections, we did not observe any differences in IFNγ levels in tears between *C*. *caviae* infected and control animals (S1 Fig). In genital chlamydia models, it has been shown that the enhancement of TNFα is also required for achieving protection against chlamydia [60, 61] and mechanisms that are nearly IFNγ independent but dependent on *Plac8* and likely on T cell degranulation exist as well [62]. Whether these mechanisms also apply to ocular chlamydial infections should be further investigated.

Our results are consistent with those of previous studies in other chlamydial animal models, which demonstrated that high infectious doses activate the immune response. In our study, low doses of *C*. *caviae* were not able to effectively trigger either the specific cellular immune response or the systemic humoral immune response. Primary ocular infection with low doses induces polyclonal activation and is likely to stimulate parts of the innate immune response. Further studies using these three distinct infectious doses are needed to elucidate the long-term immune responses and protection in repeated chlamydial infections.

## Supporting information

S1 Fig The levels of IFNγ in the tears of guinea pigs infected with a single ocular instillation of three different *C*. *caviae* doses. The amount of *C*. *caviae* applied per guinea pig eye (expressed in IFU) and screening time points within post-infection period (day 4 –dpi4, day 7 –dpi7, day 14 –dpi14, day 21 –dpi21) are indicated on the x-axis. The start of infection is considered as day 0. The age-matched non-infected guinea pigs (nc) were used as a negative control. IFNγ concentration was determined by ELISA (Cusabio Biotech, Baltimore, USA) according to manufacturer’s instructions. The statistical significance of the observed differences was evaluated using the two-way ANOVA test followed by Tukey multiple comparisons test. No statistically significant differences were recorded.
(TIF)
