# Supplementary figures and images for: The effect of infectious dose on humoral and cellular immune responses in Chlamydophila caviae primary ocular infection
Source: PLoS One. 2017 Jul 5;12(7):e0180551. doi: 10.1371/journal.pone.0180551 (PMC5498042; doi:10.1371/journal.pone.0180551)

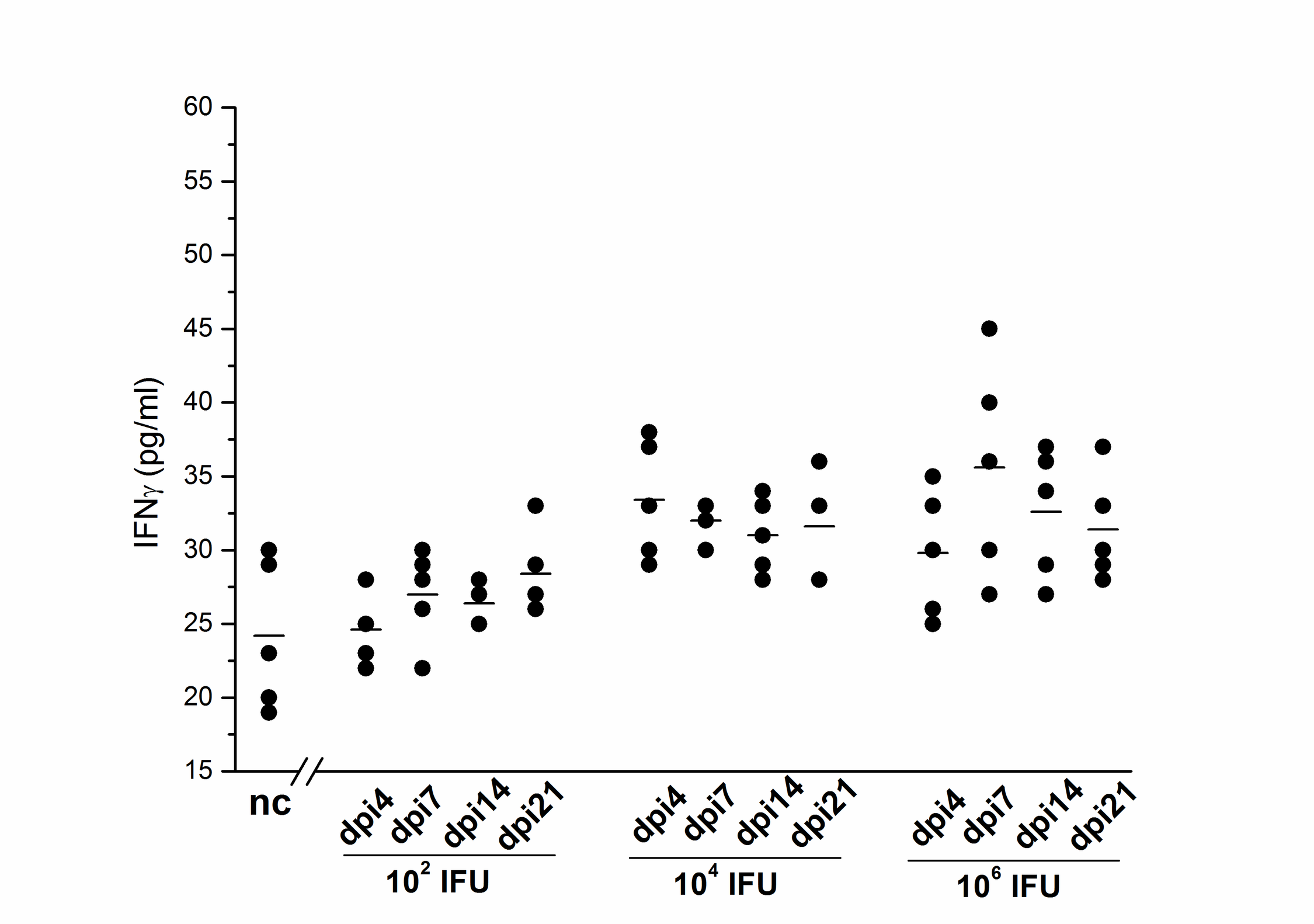

Supplement: S1 Fig — The amount of C. caviae applied per guinea pig eye (expressed in IFU) and screening time points within post-infection period (day 4 –dpi4, day 7 –dpi7, day 14 –dpi14, day 21 –dpi21) are indicated on the x-axis. The start of infection is considered as day 0. The age-matched non-infected guinea pigs (nc) were used as a negative control. IFNγ concentration was determined by ELISA (Cusabio Biotech, Baltimore, USA) according to manufacturer’s instructions. The statistical significance of the observed differences was evaluated using the two-way ANOVA test followed by Tukey multiple comparisons test. No statistically significant differences were recorded. (TIF) [file pone.0180551.s001.tif]
